# Straight-sided beer and cider glasses to reduce alcohol sales for on-site consumption: A randomised crossover trial in bars

**DOI:** 10.1016/j.socscimed.2021.113911

**Published:** 2021-06

**Authors:** Laura A. Brocklebank, Anna K.M. Blackwell, Theresa M. Marteau, Gareth J. Hollands, Paul C. Fletcher, Katie De-loyde, Richard W. Morris, Mark A. Pilling, Rachel Pechey, Olivia M. Maynard, Angela S. Attwood, Marcus R. Munafò

**Affiliations:** a School of Psychological Science, University of Bristol, 12a Priory Road, Bristol, BS8 1TU, UK; b Department of Behavioural Science and Health, University College London, 1-19 Torrington Place, London, WC1E 6BT, UK; c Behaviour and Health Research Unit, University of Cambridge, Cambridge, CB2 0SR, UK; d Department of Psychiatry, University of Cambridge, Clifford Allbut Building, Addenbrooke's Hospital, Cambridge, CB2 0SP, UK; e Cambridgeshire and Peterborough NHS Foundation Trust, Cambridge, CB21 5EF, UK; f Wellcome Trust MRC Institute of Metabolic Science, University of Cambridge, Cambridge Biomedical Campus, Cambridge, CB2 0QQ, UK; g Department of Population Health Sciences, Bristol Medical School, University of Bristol, Bristol, UK; h Nuffield Department of Primary Care Health Sciences, University of Oxford, Radcliffe Primary Care Building, Radcliffe Observatory Quarter, Woodstock Road, Oxford, OX2 6GG, UK; i National Institute for Health Research Biomedical Research Centre, University Hospitals Bristol NHS Foundation Trust and University of Bristol, Bristol, UK; j MRC Integrative Epidemiology Unit, University of Bristol, Oakfield House, Oakfield Grove, Bristol, BS8 2BN, UK

**Keywords:** Choice architecture, Nudging, Glass shape, Alcohol consumption

## Abstract

**Background:**

Straight-sided glasses can slow the rate of lager consumption in a laboratory setting compared with curved glasses. Slower drinking rates may lower overall alcohol consumption. Glass shape is therefore a potential target for intervention. The aim of this randomised crossover trial was to estimate the impact of serving draught beer and cider in straight-sided glasses, compared with usual, predominantly curved glasses, on alcohol sales for on-site consumption in bars.

**Methods:**

Twenty-four bars in England completed two intervention periods (A) and two control periods (B) in a randomised order: 1) BABA; 2) BAAB; 3) ABBA; or 4) ABAB. Each period lasted two weeks and involved serving draught beer and cider in either straight-sided glasses (A) or the venue's usual glasses (≥75% curved; B). The primary outcome was the mean volume (in litres) of draught beer and cider sold weekly, compared between A and B periods using a paired-samples *t*-test on aggregate data. A regression model adjusted for season, order, special events, and busyness.

**Findings:**

Mean weekly volume sales of draught beer and cider was 690·9 L (SD 491·3 L) across A periods and 732·5 L (SD 501·0 L) across B periods. The adjusted mean difference (A minus B) was 8·9 L per week (95% CI -45·5 to 63·3; *p* = 0·737).

**Interpretation:**

This study provides no clear evidence that using straight-sided glasses, compared with usual, predominantly curved glasses, reduces the volume of draught beer and cider sold for on-site consumption in bars.

## HIGHLIGHTS

• Straight-sided glasses do not reduce beer sales in bars versus usual glasses.
• Usual beer glasses are predominantly curved, but there are many different types.
• Other interventions may warrant attention, such as reducing serving sizes.

## Graphical abstract

**Figure:**
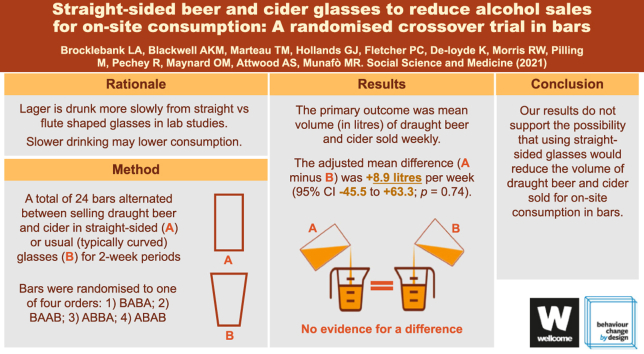

## 1 Introduction

Excessive alcohol consumption is associated with over 200 health conditions (World Health Organisation, 2018) and is among the top five risk factors for disease globally (Lim et al., 2012). It creates a substantial burden on public services, including over one million hospital admissions and £3·5 billion in costs to the UK healthcare system (National Health Service [NHS] per year) (Health & Social Care Info, 2015). Given the personal, societal, and economic burden of excessive alcohol consumption, it is unsurprising that alcohol control is high on the political agenda in many countries, including in the UK. However, reducing population levels of alcohol consumption is notoriously difficult. In a recent review of the effectiveness and cost-effectiveness of alcohol control policies, those addressing the affordability of alcohol (e.g., increasing taxation) were identified as the most successful (Burton et al., 2017). However, these measures face strong opposition from the alcohol industry, the general public, and policy makers. Furthermore, the Organisation for Economic Co-operation and Development (OECD) suggests that a coherent overall policy approach that combines the most effective and cost-effective alcohol control policies may change social norms around drinking to increase the impact on alcohol-related harm (Tackling Harmful Al, 2015). Therefore, additional alcohol control interventions should be investigated.

Interventions that alter the proximal physical micro-environments in which behaviours occurs – often called ‘choice architecture’ or ‘nudging’ interventions – hold promise for reducing unhealthy behaviours at the population level, including excessive alcohol consumption (Hollands et al., 2017). Such interventions may often require minimal conscious engagement, mainly working via autonomic or non-conscious psychological processes (Hofmann et al., 2008; Hollands et al., 2016). One aspect of the drinking environment that has the potential to influence drinking behaviour – possibly outside of awareness – is the glassware in which drinks are served. There is a growing evidence base for the effects of glass size and shape on alcohol consumption. A recent mega-analysis combining raw data from eight field studies found that serving wine in larger glasses increased wine sales for on-site consumption in restaurants (Pilling et al., 2020). There is also evidence that glass shape influences the rate of alcohol consumption under controlled laboratory conditions, with social alcohol drinkers taking, on average, 4 min longer to finish a full glass (341 ml) of lager when it was served in a straight-sided glass compared with a curved glass (Attwood et al., 2012). The underlying mechanisms are unknown, but one potential mechanism – the driving hypothesis for this study – includes perceptual bias when estimating the volume remaining in a drink (Attwood et al., 2012; Langfield et al., 2020). Overestimating the volume remaining in a drink may result in drinkers perceiving themselves as drinking more slowly than they actually are, and increasing their drinking rate accordingly. Other potential mechanisms include 1) glass shape affording different sip sizes (Langfield et al., 2020); and 2) glass shape (i.e., rounded or angular) being associated with drink taste or flavour perception (i.e., sweetness, fruitiness, and intensity) (Machiels, 2018; Mirabito et al., 2017; Spence and Van Doorn, 2017). These mechanisms are not necessarily mutually exclusive, with more than one potentially contributing to any effect. Given slower drinking rates may lower overall alcohol consumption, glass shape is a potential target as an alcohol control intervention.

The aim of this study was to estimate the impact of serving draught beer (which includes lager and ale) and cider in straight-sided glasses, compared with usual, predominantly curved glasses, on alcohol sales for on-site consumption in bars. We hypothesised that a lower volume of draught beer and cider would be sold when bars served these alcoholic drinks in straight-sided glasses compared with their usual glasses.

## 2 Methods

### 2.1 Study design

**Fig. 1:**
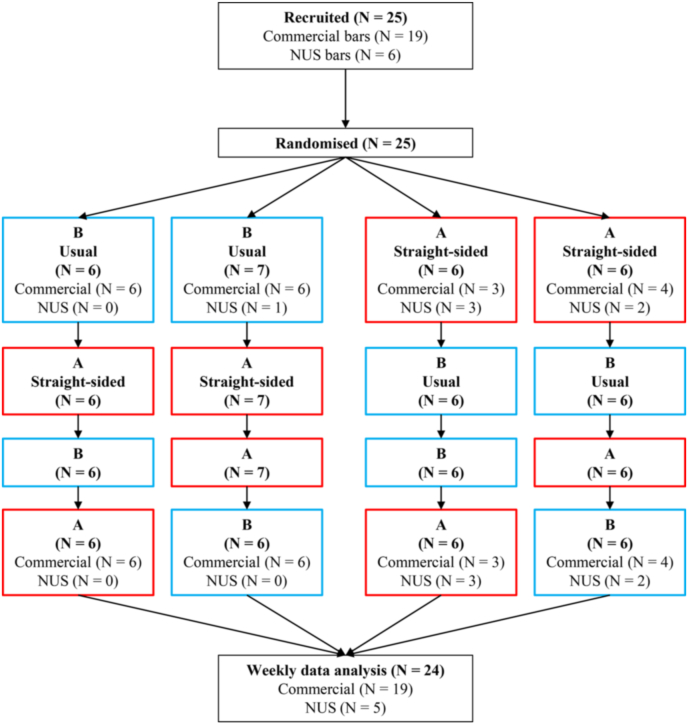
CONSORT flow diagram. NUS, national union of students.

Twenty-four bars in the UK took part in this randomised four-period crossover (i.e., multiple-treatment reversal) trial. All participating venues completed two intervention periods (A) and two control periods (B) in a randomised order. Six venues were randomised to each of four possible orders: 1) BABA; 2) BAAB; 3) ABBA; or 4) ABAB (Fig. 1). Each period lasted two weeks and therefore each venue was monitored for eight weeks in total. Draught beer and cider were served in straight-sided pint (568-ml) and half-pint (284-ml) glasses during the intervention condition and in the venue's usual pint and half-pint glasses during the control condition (i.e., usual practice). This type of intervention is categorised as a Size x Product intervention within a classification system for choice architecture interventions (Hollands et al., 2017).

### 2.2 Participants

We collected aggregate and not individual-level data, with the unit of randomisation and data collection being the bars. Either owners or managers provided written informed consent for their bar to take part in the study.

#### 2.2.1 Recruitment

We recruited a convenience sample of 25 bars. Bars were recruited by directly contacting owners/managers. This was facilitated by personal contacts, snowball sampling, and the National Union of Students (NUS) who recruited a small number of bars (N = 6, including one drop-out). Bar owners were offered financial compensation of £500 for their time participating in this study. They were also given the option to keep the straight-sided glassware for use after the study. Six NUS bars were recruited in Lampeter (Wales), Edinburgh, Kent, Salford, Bristol, and Sheffield, and nineteen commercial bars were recruited in Bristol (N = 13), Stroud (N = 3), Bury St Edmunds (N = 1), Cheltenham (N = 1), and Cardiff (N = 1). Data collection took place between October 2018 and September 2019, with bars starting the eight-week trial when it was convenient for them. This included the Christmas period, when alcohol consumption is likely to be higher than normal, for only one of the venues.

#### 2.2.2 Eligibility criteria

In order to take part in this study, bars had to meet the following inclusion criteria:

1. Sell more than 160 pints, or 90 L, of draught beer and cider (combined) per week.
2. Approximately 75% or more of their usual pint glasses are curved. Glasses were defined as ‘straight-sided’ if the sides were parallel, but ‘curved’ otherwise.
3. Their licensing conditions will allow them to serve draught beer and cider in straight-sided pint and half-pint glasses during the intervention condition.
4. Have an electronic point of sale (EPOS) till system, or an equivalent, to record itemised sales for all drinks, including draught beer and cider.

#### 2.2.3 Sample size calculation

Following a feasibility study in 3 bars (Troy et al., 2015), we estimated that a total of 24 bars would be required to allow an effect size of 0.6 for monetary takings to be detected between the two conditions (intervention [straight-sided glassware] and control [usual glassware]), with at least 90% power and an alpha level of 5% for an F-test. This calculation assumed a within-venue correlation coefficient of *r* = 0.65 between periods across the two conditions. In the study by Troy et al. (2015), one standard deviation (SD) equated to 27% of monetary takings across the three participating venues (Troy et al., 2015). Therefore, using monetary takings as an approximate proxy for volume sold, the current study was designed to detect a mean difference in volume sold of at least 16% between the two conditions.

When finalising the design of the current study, we elected to use volume sales of draught beer and cider as our primary outcome, as this is a more proximal measure of the actual behaviour we are interested in (i.e., consumption of draught beer and cider) than total monetary takings for all drinks. However, our original sample size calculation remains valid, as weekly volume sales of draught beer and cider and weekly monetary takings for all drinks were highly correlated (r = 94.5%) for the sub-sample of 3 bars that provided these data (calculated from 23 data points using a generalised linear model to account for the different number of repeated measures across the 3 bars).

#### 2.2.4 Randomisation and masking

The random order for the four periods was determined at the start of the study using a computer-generated list of random numbers, which was produced in Stata 15 for 24 bars before recruitment had begun by RWM who was not involved with subsequent data handling or analysis. Blocked randomisation was used to ensure that an equal number of venues (N = 6) were assigned to each of four possible orders (Fig. 1). The order was concealed until after the bar owner/manager had agreed to the study protocol. Due to the nature of the study, it was not possible to blind the research team or the participating venues to order allocation, but the statistician conducting the analysis was blinded. Upon request of the research team, bar staff attempted to blind drinkers to the study hypothesis (see section on *Study protocol,* below, for more details).

## 3 Materials

Venues served draught beer and cider in either their usual pint and half-pint glasses (control condition) or straight-sided pint and half-pint glasses provided by the research team (intervention condition).

### 3.1 Usual glassware

Beyond the glass shape restrictions described in the eligibility criteria, no other restrictions were placed on the venues' usual glassware. The different glass shapes are shown in Supplementary Table S1, with the ‘Weizen’ glass shape (i.e., much wider at the top than at the bottom of the glass) matching the curved glassware used to inform the hypothesis for this study (Attwood et al., 2012; Troy et al., 2015). An objective measure of the degree of curvature is the midpoint bias – the difference in centimetres (cm) between the midpoint in terms of height and the midpoint in terms of volume. A perfectly straight-sided glass would have a midpoint bias of zero. For each venue, the following information was recorded for each type of pint glass used during the control condition: 1) shape; 2) midpoint bias; and 3) proportion of total pint glasses.

### 3.2 Straight-sided glassware

Venues were given the option of two different types of straight-sided glassware, both with parallel sides:

1. Highball pint and half-pint glasses, with the option of a nucleated or non-nucleated base (Fig. 2a).

**Fig. 2:**
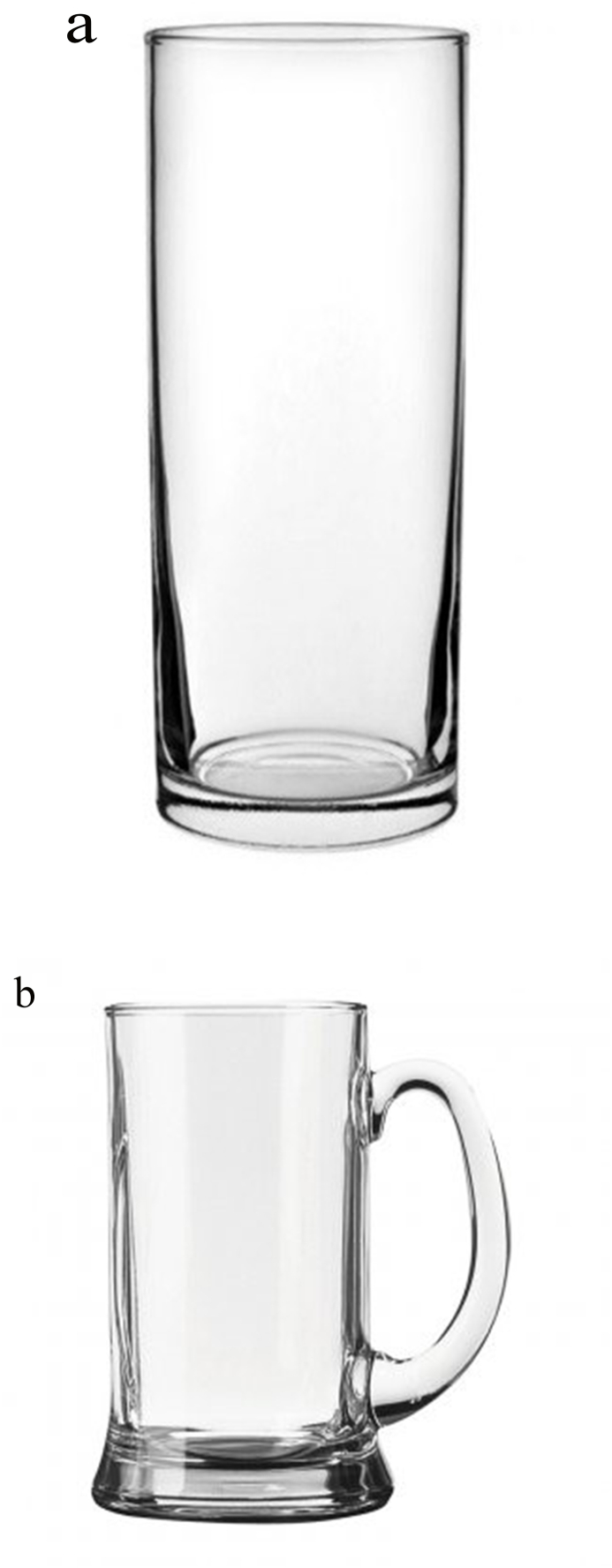
Straight-sided glassware used during the intervention condition: highball pint and half-pint glasses (left) and/or pint and half-pint tankards (right). Both types of glassware had parallel sides.
2. Pint and half-pint tankards (Fig. 2b), which were requested by some owners/managers specifically for ale drinkers.

Venues were provided with enough straight-sided glasses to replace their usual glasses, and this could consist of highball glasses only, tankards only, or both (40%, 4%, and 56% of venues, respectively).

## 4 Measures

At the end of the eight-week study period, all relevant till data were sent to the research team and used to derive the following outcome variables.

### 4.1 Primary outcome

The primary outcome for this study was the mean volume (in litres) of draught beer and cider sold weekly. This was an aggregated value of the two 14-day A periods and the two 14-day B periods, respectively, expressed as a weekly average.

### 4.2 Secondary outcomes

Lager, ale, and cider were examined separately to explore whether the effect of the intervention differed depending on the type of draught drink. Furthermore, the effects of the intervention on the consumption of other alcoholic (i.e., bottled beer, wine, and spirits) and non-alcoholic drinks (soft and alcohol-free drinks) were examined to explore whether the introduction of straight-sided glassware for draught drinks influenced drinkers’ selection of other types of drink (e.g., as a result of not wishing to use the straight-sided glasses). All secondary outcomes were aggregated values of the two 14-day A periods and the two 14-day B periods, respectively, expressed as a weekly average.

### 4.3 Tertiary outcome

Seventeen venues also provided more detailed daily data on the mean volume (in litres) of draught beer and cider sold.

### 4.4 Additional measures

If drinkers left the participating venues early to go for another drink elsewhere (e.g., due to not wishing to use the straight-sided glasses), a reduction in volume sales may be erroneously interpreted as customers drinking less rather than there being fewer customers. Therefore, as a measure of busyness, a head count of customers was collected once for each period (i.e., four times in total for each venue). Head counts were completed by an independent mystery shopper agency (Mystery Shoppers Limited, www.mystery-shoppers.co.uk) in conjunction with fidelity checks (i.e., to check if the correct glassware [usual or straight-sided] was being used). These visits took place between 7 p.m. and 10 p.m. on a Friday or Saturday, with both the day and time remaining consistent across all four periods for each venue. Data were also collected on the total number of special events during each period that were likely to increase alcohol sales, excluding regular (i.e., weekly or fortnightly) events. Finally, two measures were used to account for between-venue variation in usual glassware: 1) weighted mean midpoint bias (weighted by the proportion of total pint glasses made up by each glass type) and 2) percentage of pint glasses that were ‘Weizen’ in shape (Supplementary Table S1).

### 4.5 Study protocol

A member of the research team visited each venue to confirm its eligibility for the study. This included photographing and measuring the midpoint bias of each type of pint glass currently in use. If eligible, either the owner or the manager was asked to provide written informed consent for their bar to take part in the study and a start date was arranged. Before the start date, the researcher revealed the order of the four periods and discussed the logistics of glassware delivery and exchanges (from usual to straight-sided and vice versa) with the bar manager.

During both intervention periods, draught beer and cider were served in straight-sided pint and half-pint glasses for two weeks. If customers asked about the change of glassware, bar staff were instructed to follow a standardised script in an attempt to blind drinkers to the study hypothesis: *“We are taking part in a research study for eight weeks, the details of which will be revealed after study completion so as not to affect the results”*. The research team provided the straight-sided glassware and bar owners/managers were given the option to keep it for use after the study. During both control periods, draught beer and cider were served in the venue's usual pint and half-pint glasses for two weeks.

Bar managers received a telephone reminder at least 24 h before each new period and the research team helped with the glassware exchange if needed. If necessary (e.g., due to space limitations), external storage was arranged for the glassware not currently in use (i.e., usual glassware during the intervention periods or straight-sided glassware during the control periods). At the end of the study, the bar manager sent the research team all relevant till data for the eight-week study period, as well as a list of all special events. As soon as all data were received, the bar was transferred £500 to compensate for their time taking part in the study.

## 5 Data analysis

### 5.1 Primary analysis

For the primary analysis of the primary outcome, mean weekly volume sales of draught beer and cider was compared between the intervention (A) periods and the control (B) periods using a paired-samples *t*-test. This analysis was repeated for all secondary outcomes. Data are reported as unadjusted mean differences with 95% confidence intervals (95% CIs) and *p* values. Model diagnostics were checked and were satisfactory.

### 5.2 Secondary analysis

For the secondary analysis of the primary outcome, a general linear mixed model was used to compare mean weekly volume sales of draught beer and cider between the intervention (A) periods and the control (B) periods after adjustment for: season at the start of Period 1; order; special events; and busyness (i.e., head count of customers). This analysis was repeated for all secondary outcomes. Two interaction terms – glass shape by season and glass shape by order were added simultaneously to the secondary analysis models, but were subsequently removed as there was no evidence of either interaction in any model. Data are reported as adjusted mean differences with 95% CIs and *p* values. Model diagnostics (residuals) were satisfactory.

### 5.3 Tertiary analysis

For the 17 venues that additionally provided aggregate data at the day level, a GAMLSS (Generalised Additive Model for Location, Scale and Shape) regression model with a skewed-t distribution (Stasinopoulos et al., 2017) was used to predict daily volume sales of draught beer and cider from glass shape, with usual glassware (i.e., the control [B] periods) being the reference group. This sample size provided approximately 80% power to detect the anticipated effect size. A GAMLSS model with a skewed-t distribution was used to account for heteroscedasticity (in venue and day of the week) and non-normality, respectively. Venue was clustered within order, and these were fitted as random effects. The model adjusted for season, day of the week, local temperature at 5 p.m. recorded by the Met Office (if available, else nearest time to 5 p.m.), and venue busyness (i.e., the total number of non-draught drinks sold, including bottled beer, wine, spirits, alcopops, soft drinks, and alcohol-free drinks). Data are reported as adjusted mean difference with 95% CI and *p* value. Model diagnostics (residuals and worm plots) were satisfactory. One known outlier (a special event) was excluded from one venue, though this did not change model conclusions.

### 5.4 Per-protocol analysis

For the per-protocol analysis, the primary and secondary analyses of the primary outcome were repeated after excluding the nine venues that failed at least one fidelity check.

### 5.5 Exploratory analyses

To examine whether the effect of the intervention differed between commercial and NUS bars, the primary and secondary analyses of the primary outcome were repeated after adding an interaction term – glass shape by bar type – to the models (pre-specified analyses). There were many different types of glass used by the participating venues during the control periods (i.e., usual glassware). Therefore, to explore whether the effect of the intervention differed according to the degree of curvature of the venue's usual glassware, the primary analysis of the primary outcome was repeated with the addition of two interaction terms, which were independently added to the model: 1) glass shape by weighted mean midpoint bias and 2) glass shape by percentage that were Weizen in shape (*post hoc* analyses).

Further details of the study methods can be found in the pre-registered study protocol on ISRCTN (ISRCTN10456720) and the Open Science Framework (OSF; https://osf.io/4kurx/). The statistical analysis plan was also pre-registered on the OSF in advance of data analysis.

In response to reviews, we also conducted a *post hoc* analysis to explore whether the effect of the intervention differed according to the percentage of intervention pint and half-pint glasses that were highball glasses or tankards, and there was no evidence that the effect of the intervention was modified by these (*p* values > 0.49).

### 5.6 Role of the funding source

The funder of the study had no role in study design, data collection, data analysis, data interpretation, or writing of the report. The corresponding author had full access to all the data in the study and had final responsibility for the decision to submit for publication.

## 6 Results

### 6.1 Sample characteristics

Twenty-five bars were recruited to this study (19 commercial and six NUS bars). One NUS bar dropped out after Period 3 of the study without giving a reason, leaving 24 bars (19 commercial and five NUS bars) that completed the study and therefore were included in the primary and secondary analyses (see Fig. 1 for the CONSORT Flow Diagram). Two bars had missing data for one week each (both during intervention periods), resulting in 190 of a possible 192 weeks’ worth of data for the primary outcome (i.e., weekly volume sales of draught beer and cider). For these 2 bars, mean weekly volume sales of draught beer and cider during the intervention (A) periods was calculated from the remaining three data points.

Seventeen bars (13 commercial and four NUS bars) additionally provided daily data. Four bars were closed on some days during the study, usually on a Monday (23 days in total; 11 days during intervention [A] periods and 12 days during control [B] periods), and another bar had missing data for seven days during an intervention period. This resulted in 922 of a possible 952 days’ worth of data for the tertiary outcome (i.e., daily volume sales of draught beer and cider). One extreme outlier was identified in these data and was subsequently removed as it was due to a known special event that greatly increased alcohol sales. However, including this outlier did not change the overall conclusions of the study.

### 6.2 Weekly volume sales

#### 6.2.1 Primary outcome

**Fig. 3:**
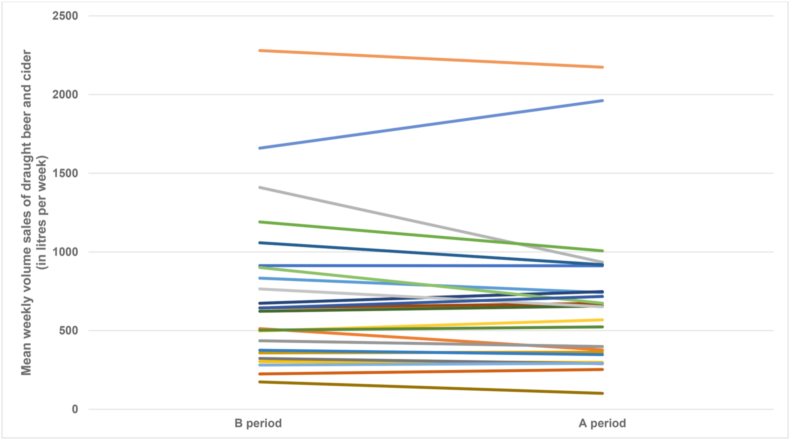
Difference in mean weekly volume sales of draught beer and cider between the intervention (A) periods using straight-sided glassware and the control (B) periods using usual glassware by participating venue. The different coloured lines represent different venues.

Mean weekly volume sales of draught beer and cider was 690·9 L (SD 491·3 L) across the intervention (A) periods and 732·5 L (SD 501·0 L) across the control (B) periods. Raw values for each participating venue are shown in Fig. 3. There was no clear evidence against the null hypothesis of glass shape (i.e., straight-sided [A] or usual [B]) having no effect. The unadjusted mean difference (A minus B) was −41·6 L per week (95% CI -101·3 to 18·0; *p* = 0·162), equivalent to a 6% reduction, and after adjustment for season, order, special events, and busyness was 8·9 L per week (95% CI -45·5 to 63·3; *p* = 0·737), equivalent to a 1% increase (Table 1). Tests for interaction of glass shape by season and glass shape by order gave *p* = 0·871 and *p* = 0·849, respectively, and therefore these interaction terms were omitted from the final models.

**Table 1:** Differences in mean weekly volume sales between the intervention (A) periods using straight-sided glassware and the control (B) periods using usual glassware.

		Period A	Period B	Unadjusted difference (A-B)		Adjusted difference (A-B)^a^	
	**N**	Raw mean (SD)	Raw mean (SD)	MD (95% CI)	*P* value	MD (95% CI)	*P* value
**Draught beer**^b^**and cider** (litres/week)	24	690·9 (491·3)	732·5 (501·0)	−41·6 (−101·3, 18·0)	0·162	8·9 (−45·5, 63·3)	0·737
**Draught lager** (litres/week)	20	329·8 (305·7)	332·9 (280·2)	−3·1 (−32·6, 26·4)	0·828	26·8 (1·9, 51·6)	0·036
**Draught ale** (litres/week)	20	256·1 (242·7)	283·0 (270·5)	−26·9 (−52·7, −1·1)	0·042	−1·0 (−27·5, 25·6)	0·941
**Draught cider** (litres/week)	21	142·9 (144·1)	149·7 (137·4)	−6·8 (−26·2, 12·7)	0·477	10·4 (−7·7, 28·5)	0·244
**Bottled beer** (total number/week)	17	64·3 (52·8)	66·6 (59·1)	−2·3 (−8·7, 4·0)	0·444	0·2 (−6·2, 6·7)	0·940
**Wine** (litres/week)	24	29·1 (30·1)	28·9 (27·6)	0·2 (−2·5, 2·9)	0·893	1·2 (−1·6, 4·0)	0·364
**Spirits** (litres/week)	24	7·0 (7·2)	7·5 (6·3)	−0·5 (−1·5, 0·5)	0·340	−0·1 (−1·2, 1·0)	0·836
**Soft drinks** (total number/week)	24	357·6 (337·9)	392·3 (346·0)	−34·8 (−87·8, 18·2)	0·188	−17·0 (−82·9, 48·8)	0·594
**Alcohol-free drinks** (total number/week)	6	10·0 (14·7)	10·0 (13·4)	0·0 (−3·8, 3·8)	1·000	1·6 (−3·7, 7·0)	0·441

a Adjusted for the season at the start of Period 1; order (BABA, BAAB, ABBA or ABAB); the total number of special events in each period; and the head count of customers in each period (as a proxy for busyness).

b Draught beer includes lager and ale. SD, standard deviation; MD, mean difference; 95% CI, 95% confidence interval.

### 6.3 Secondary outcomes

Similar results were observed for all secondary outcomes – there was no clear evidence against the null hypothesis of glass shape having no effect (Table 1).

### 6.4 Per-protocol analysis

Nine of 24 bars (38%) failed at least one fidelity check. All nine bars failed for using a mixture of correct and incorrect glassware, rather than for exclusively using incorrect glassware (i.e. usual glassware during the intervention [A] periods or straight-sided glassware during the control [B] periods). However, excluding these bars did not change the conclusions of the study (adjusted mean difference [95% CI] 31·2 L per week [-32·9 to 95·3]; *p* = 0·310).

### 6.5 Exploratory analyses

There was no clear evidence that the effect of the intervention differed according to the type of bar (*p* = 0·727 for glass shape by bar type). There was also no clear evidence that the effect of the intervention differed according to the degree of curvature of the bar's usual glassware (*p* = 0·520 for glass shape by weighted mean midpoint bias and *p* = 0·160 for glass shape by percentage that were Weizen in shape).

### 6.6 Daily volume sales

#### 6.6.1 Tertiary analysis

The more detailed daily data provided concordant results, with an effect size that was very similar to the weekly analysis. The mean difference in draught beer and cider consumption (A minus B) after adjustment for season, day of the week, temperature, and venue busyness was 1·4 L per day (95% CI -0·8 to 3·6; *p* = 0·218), which is equivalent to an increase of 9·8 L per week or 2%.

## 7 Discussion

Contrary to our hypothesis, in this randomised crossover trial we found no clear evidence that serving draught beer and cider in straight-sided pint and half-pint glasses reduced the weekly or daily volume of draught beer and cider sold in bars, compared with usual pint and half-pint glasses that were predominantly (i.e., 75% or more) curved.

Pre-existing evidence of the effect of glass shape on alcohol consumption is based on one laboratory study of students conducted in 2012. Attwood et al. (2012) found that straight-sided glasses, compared with curved glasses, reduced the rate of lager consumption (Attwood et al., 2012). This study has yet to be replicated. In a series of laboratory studies, Langfield et al. (2020) found that straight-sided glasses, compared with outward-sloped glasses, reduced the volume of sugary drinks consumed (Langfield et al., 2020). They also identified a potential mechanism for reduced consumption – straight-sided glasses elicit greater pursing of the lips, which in turn reduces sip size.

There are a number of possible reasons for the differences in results between these laboratory studies and the current study. First, the laboratory studies provided both the straight-sided and curved glassware, and there was a substantial difference between the two glass shapes. For example, Attwood et al. (2012) compared straight-sided beer glasses with Weizen beer glasses – i.e., much wider at the top than at the bottom of the glass (Supplementary Table S1). (Attwood et al., 2012) In contrast, the curved glassware in the current study was the bars' usual glassware. On average, 42% of the usual pint glasses used during the control periods were ‘Shaker’ in shape – i.e., only slightly wider at the top than at the bottom of the glass (Supplementary Table S1). Therefore, one possible reason why an effect of glass shape on alcohol sales for on-site consumption was not observed in the current study is that the straight-sided intervention glassware was too similar to the usual glassware. Second, the size of the glasses differed – pint (568-ml) and half-pint (284-ml) beer glasses were used in this study, compared with 341-ml beer glasses (Attwood et al., 2012) and 165-ml wine glasses (Langfield et al., 2020). Third, other variables that are present only in field settings – e.g., the social context and the drinking rates of others – might have had a larger effect than that of glass shape, thereby masking any effect of glass shape. Finally, slower drinking rates will not lower overall alcohol consumption if drinkers base the length of their stay in a bar on the number of drinks consumed rather than the amount of time.

### 7.1 Strengths and limitations

The current study is novel, being the first – to our knowledge – to examine the impact of glass shape on alcohol sales for on-site consumption in a real-world drinking environment – *i.e.* bars. It also included a large number of venues, with 24 bars completing the eight-week trial. Nevertheless, several limitations should be noted. First, the outcome measure was volume sales for on-site consumption rather than consumption itself. However, people generally consume most alcohol they purchase, with other studies suggesting that wastage is likely to be low (Kerr and Long, 2010; Kersbergen et al., 2018; ealth Scotland. Moni, 2012). For example, in the study by Kersbergen et al. (2018), less than 1% of wine purchased in a bar setting was left undrunk (Kersbergen et al., 2018). Volume sales is therefore a valid measure of consumption when purchased for immediate consumption, such as in a bar or restaurant.

Second, many different types of glass were used by the participating venues during the control periods (i.e., usual glassware), with different shapes and therefore different amounts of curvature. The most common glass shape was ‘Shaker’ (Supplementary Table S1). These glasses were only slightly wider at the top than at the bottom of the glass and therefore did not differ substantially from the straight-sided intervention glasses. However, the current study was not intended to be a direct replication of the laboratory study by Attwood et al. (2012) (Attwood et al., 2012); it was designed to inform policy by providing evidence that could be used to guide decisions around whether particular glass shapes should be changed from what is typically used currently. Therefore, using usual glassware as the control was important for estimating the impact of changing existing glassware. Nevertheless, *post hoc* analyses were conducted to explore whether the effect of the intervention differed according to characteristics of the usual glassware (i.e., shape and curvature), which could provide information about possible mechanisms. There was no evidence of differential effects by any of the characteristics. Most usual pint glasses were branded, whereas none of the straight-sided intervention glasses were branded. Therefore, any differences between the two conditions could be due to branding effects rather than glass shape effects. However, the extent to which this may play a role is currently unclear, given – to our knowledge – no studies to date have examined the impact of glassware branding on alcohol consumption. Other glass design characteristics that influence drink enjoyment might in principle also influence consumption, although a recent experimental study found no clear evidence that nucleation influenced lager consumption (either in terms of volume consumed or drinking rate) despite increasing its visual appeal (Troy et al., 2019).

Finally, the majority of the participating venues were independent bars in South West England. This may limit the generalisability of the findings to other regions of the UK, to other countries that have different drinking cultures, or to other licensed premises where drinking behaviour may be different. Furthermore, five of the venues were student bars. Hazardous drinking is more common among students than the general population (Attwood et al., 2012), limiting the generalisability of the findings to less frequent drinkers. However, we found no clear evidence that the effect of the intervention differed between non-student and student bars.

### 7.2 Implications for research and practice

One possible reason why an effect of glass shape on alcohol sales for on-site consumption was not observed in the current study is that the straight-sided intervention glasses were too similar to the usual glasses. This could be tested in a future study in which both the straight-sided and curved glassware are provided to ensure that the difference between the two glass shapes is greater. Should further research determine with more certainty that glass shape does not modify alcohol consumption, other potentially more promising choice architecture interventions for reducing consumption may warrant attention. This includes those targeting the size of glassware (e.g., small vs. large wine glasses) and serving sizes (e.g., introducing a two-third pint option for draught beer and cider, or removing the largest serving size for a glass of wine [usually 250-ml]). A recent mega-analysis found that the volume of wine sold in restaurants was, on average, 7·3% higher when 370-ml wine glasses were used compared with 300-ml wine glasses (Pilling et al., 2020). Furthermore, Kersbergen et al. (2018) found that reducing the serving sizes of beer and wine reduced alcohol consumption in both a laboratory and a more naturalistic setting, although this was only assessed within a single drinking occasion – i.e., at a quiz event in a private room of a bar (Kersbergen et al., 2018). Draught beer and cider are normally served to fill the glass, but this is rarely the case for wine. Further research could therefore assess the effect of wine glass shape on wine consumption.

## 8 Conclusion

This study provides no clear evidence that serving draught beer and cider in straight-sided glasses, compared with usual, predominantly curved glasses, reduces the volume of draught beer and cider sold for on-site consumption in bars.

## Credit author statement

LAB was responsible for study design, data collection, data interpretation, and manuscript writing. AKMB was responsible for study design, data collection, data interpretation, and manuscript review. KDL and MAP were responsible for study design, data analysis, data interpretation, and manuscript review. TMM, GJH, PCF, RWM, RP, OMM, ASA, and MRM were responsible for study design, data interpretation, and manuscript review.

## Ethics approval

Approved by the Faculty of Science Research Ethics Committee at the University of Bristol (approval code: 73,621).

## Data sharing

Data are available at the University of Bristol data repository, data.bris, at https://data.bris.ac.uk/data/dataset/3lvmtuw50swi329zj45b2isija.

## Declaration of competing interest

We declare no competing interests.
