# Supplementary material for: Straight-sided beer and cider glasses to reduce alcohol sales for on-site consumption: A randomised crossover trial in bars
Source: Soc Sci Med. 2021 Jun;278:113911. doi: 10.1016/j.socscimed.2021.113911 (PMC8146727; doi:10.1016/j.socscimed.2021.113911)
Supplement: Multimedia component 1 [file mmc1.docx]

**Supplementary Appendix**

**Supplementary Table S1. Percentage of usual pint glasses made up by each glass shape for each venue.**

|  | 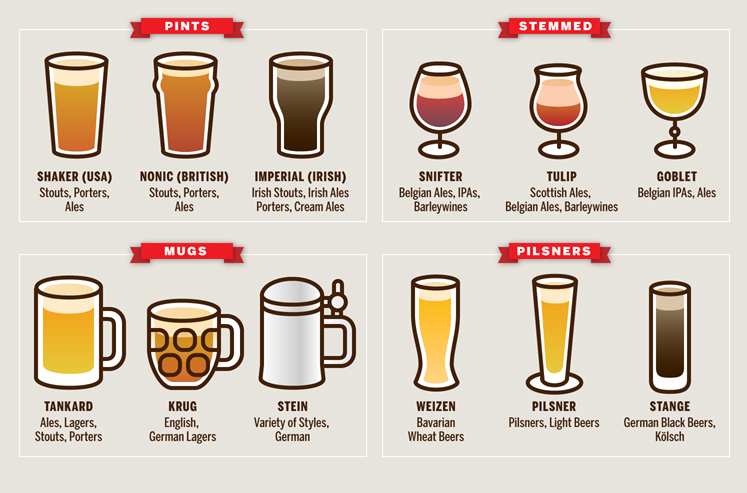 | 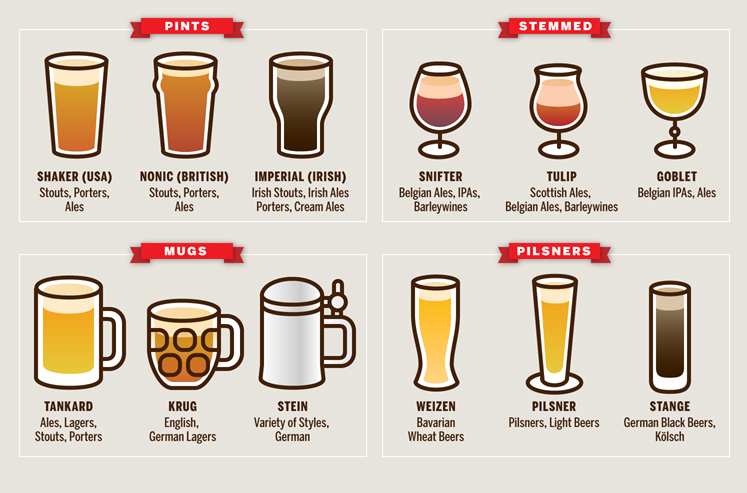 | 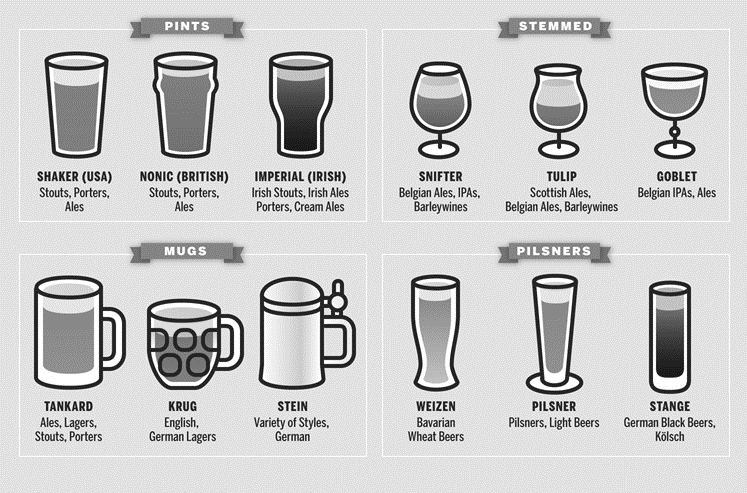 | 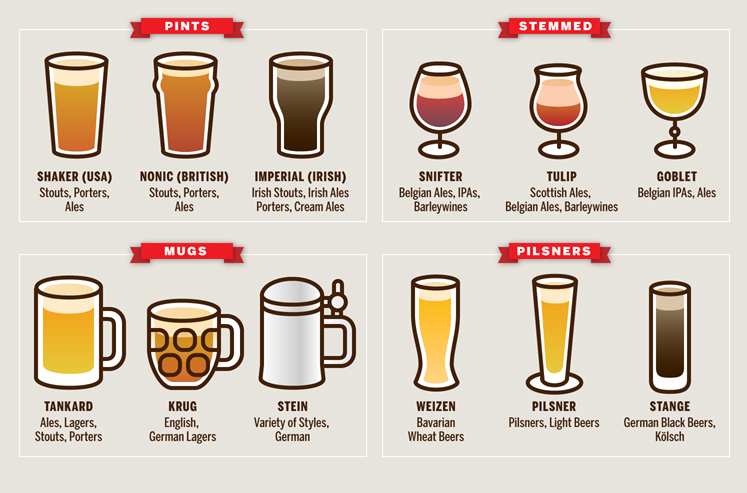 | 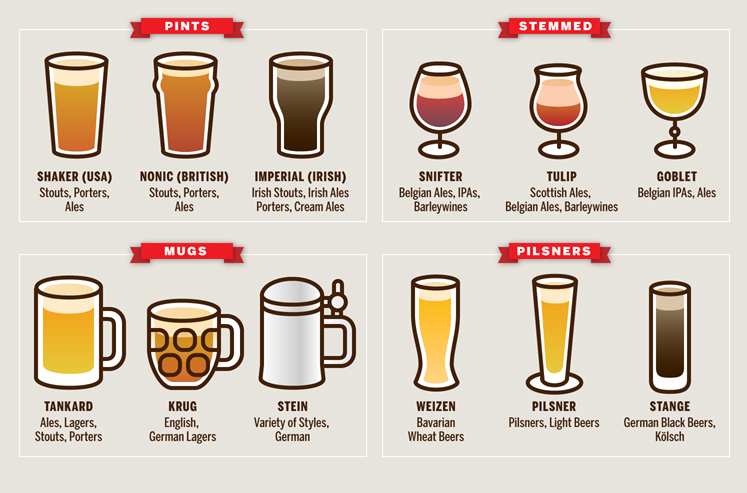 | 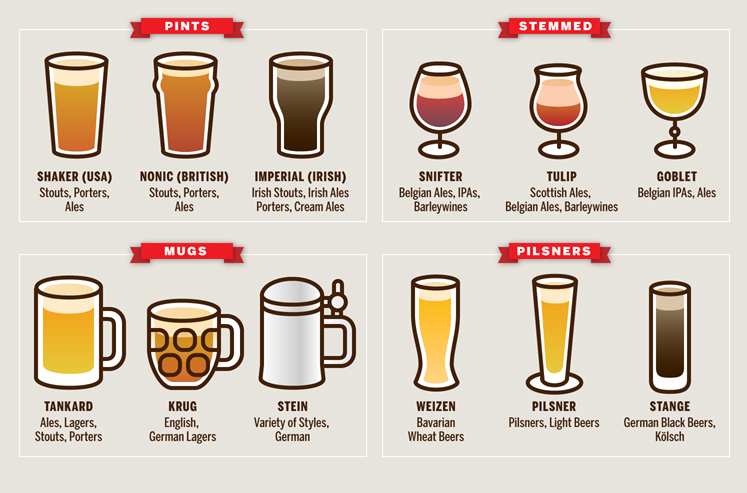 | 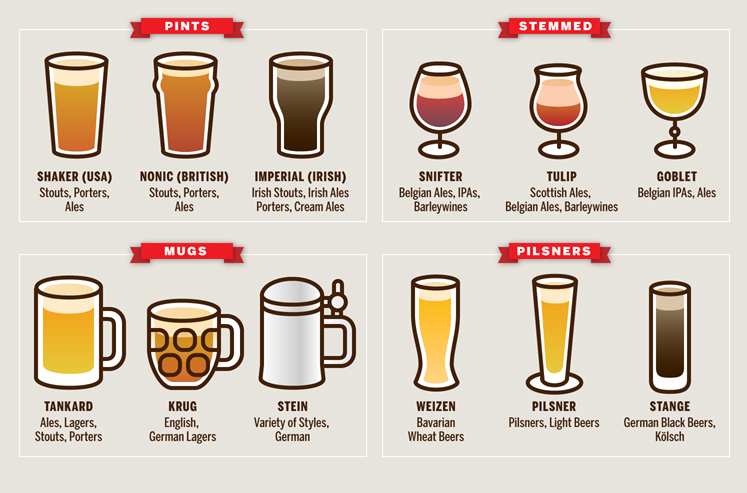 |
| --- | --- | --- | --- | --- | --- | --- | --- |
| **Bar ID** | **Shaker** | **Nonic** | **Imperial** | **Stemmed** | **Tankard** | **Krug** | **Weizen** |
| 56 | 0 | 50 | 16·7 | 0 | 0 | 0 | 33·4 |
| 57 | 40 | 20 | 20 | 0 | 20 | 0 | 0 |
| 61 | 95 | 0 | 0 | 0 | 0 | 0 | 5 |
| 63 | 38·8 | 0·4 | 39·2 | 0 | 0 | 0 | 21·6 |
| 64 | 48·4 | 0 | 21·7 | 25 | 0 | 5 | 0 |
| 65 | 98 | 0 | 0 | 0 | 0 | 2 | 0 |
| 66 | 10 | 0 | 0 | 22·5 | 0 | 22·5 | 45 |
| 70 | 35·6 | 0 | 31·2 | 5·6 | 11·2 | 0 | 16·8 |
| 71 | 35 | 0 | 0 | 0 | 5 | 5 | 55 |
| 72 | 0 | 90 | 2 | 0 | 0 | 0 | 8 |
| 74 | 60 | 0 | 3·3 | 3·3 | 0 | 0 | 33·3 |
| 77 | 35 | 0 | 10 | 0 | 0 | 5 | 50 |
| 78 | 100 | 0 | 0 | 0 | 0 | 0 | 0 |
| 84 | 0 | 0 | 70 | 0 | 0 | 0 | 30 |
| 86 | 0 | 75 | 0 | 10 | 10 | 2·5 | 2·5 |
| 88 | 70 | 0 | 0 | 0 | 0 | 0 | 30 |
| 91 | 43·4 | 0 | 0 | 19·3 | 0 | 0 | 37·3 |
| 92 | 80 | 0 | 0 | 0 | 0 | 0 | 20 |
| 95 | 0 | 0 | 17 | 4 | 9 | 0 | 70 |
| 96 | 60 | 0 | 0 | 0 | 0 | 0 | 40 |
| 98 | 0 | 0 | 50 | 0 | 0 | 0 | 50 |
| 99 | 50 | 20 | 22·5 | 0 | 0 | 7·5 | 0 |
| 101 | 50 | 0 | 0 | 0 | 50 | 0 | 0 |
| 102 | 50 | 0 | 0 | 0 | 0 | 0 | 50 |
| **Mean** | **41·6** | **10·6** | **12·7** | **3·7** | **4·4** | **2·1** | **24·9** |

The most common glass shape (or shapes in the case of equal percentages) for each venue are highlighted in grey.

Eight possible glass shapes were used to categorise the usual pint glasses in this study.^1^ All of these glass shapes fall under the definition of ‘curved’, except for the ‘Tankard’ shape, which is the same width from the top to the bottom of the glass. The ‘Weizen’ shape (i.e., much wider at the top than at the bottom of the glass) matches the curved glassware used to inform the hypothesis for this study.^2,3^

**References**

1. Daily Infographics. Different Types of Pint Glasses. Mar 9, 2015. <http://dailyinfographics.eu/different-types-of-pint-glasses/> (accessed Jul, 2020)

2. Attwood AS, Scott-Samuel NE, Stothart G, Munafò MR. Glass shape influences consumption rate for alcoholic beverages. *PloS one* 2012; **7**: e43007

3. Troy DM, Maynard OM, Hickman M, Attwood AS, Munafò MR. The effect of glass shape on alcohol consumption in a naturalistic setting: a feasibility study. *Pilot and Feasibility Studies* 2015; **1**: 27
